# Anogenital Distance: Defining “Normal”

**DOI:** 10.1289/ehp.114-a399a

**Published:** 2006-07

**Authors:** Bernard Weiss

**Affiliations:** University of Rochester, Rochester, New York, E-mail: bernard_weiss@urmc.rochester.edu

In their letter to *EHP*, McEwen and Renner (2006) dismissed the findings of Swan et al. (2005), who reported a significant relationship between a measure of anogenital distance (AGD) in boys and levels of phthalate metabolites in their mothers’ urine during pregnancy. AGD is a sexually dimorphic index that, on average, is twice as great in males as in females, so it serves as a marker of proper male development. McEwen and Renner based their argument on an idiosyncratic form of logic. They asserted that

All male infants evaluated in the study appeared normal … there is no evidence for potential adverse effect in the test population. … no conclusion can be drawn whether the reported values are normal or abnormal. The range of AGD values … likely represents typical biologic variation that would be expected to occur among normal study subjects.

McEwen and Renner seem to be wholly unfamiliar with the meaning of a modest or even a slight shift in the mean of an index that reflects the distribution of susceptibility in a population. I have pointed out (Weiss 1988) that even a 5-point (5%) reduction in mean intelligence quotient in a population of 100 million increases the number of individuals classified as retarded from 6 million to 9.4 million. It is this kind of relationship that eventually prompted the Centers for Disease Control and Prevention (CDC) to lower its definition of elevated lead risk levels in blood, set at 40 μg/dL in 1970, to 10 μg/dL in 1991 (CDC 1991). Bellinger (2006) put it this way:

A small change in the mean signals predictable accompanying changes in the proportions of individuals in the source population who fall into the tails of the distribution, where individuals who meet diagnostic criteria are found. Thus, the importance of a shift in group mean lies not in what it indicates about the average change among members of the study sample, but what it implies about the changes in the tails of the distribution in the population from which the study sample was drawn.

He noted, based on Rose (1981), that in a population with a prevalence of clinically defined hypertension of 15%, a 5-mm reduction in mean systolic blood pressure would result in a 33% decrease in prevalence (Bellinger 2006). Epidemiologists recognize that a slight decrease in mean blood pressure in a population is translated into a major decrease in the incidence of serious cardiovascular events such as heart attacks.

We already know that shortened AGD at birth is one element, the leading edge, as it were, of the “phthalate syndrome” in rats, which is marked by testicular pathology, reduced spermatogenesis, hypospadias, and cryptorchidism, a compilation of signs indicating disordered male development that Sharpe (2001) and others have noted to be on the increase in industrialized nations. An almost imperceptible shift to a lower mean AGD in the human male would foreshadow a heightened prevalence of reproductive system dysfunction. Is that the connection now emerging in the clinic?

If McEwen and Renner’s (2006) criteria for “normal” were to govern the way in which we define the health risks of lead exposure, we would be basing our criteria on the number of children brought into hospital emergency rooms with lead poisoning rather than on the threats it poses to their neurobehavioral development. No parent, and no community, would tolerate such a definition these days.
